# PUM1 Promotes Tumor Progression by Activating DEPTOR‐Meditated Glycolysis in Gastric Cancer

**DOI:** 10.1002/advs.202301190

**Published:** 2023-07-19

**Authors:** Songcheng Yin, Huifang Liu, Zhijun Zhou, Xiaoyu Xu, Pengliang Wang, Wei Chen, Guofei Deng, Han Wang, Hong Yu, Liang Gu, Mingyu Huo, Min Li, Leli Zeng, Yulong He, Changhua Zhang

**Affiliations:** ^1^ Digestive Diseases Center Guangdong Provincial Key Laboratory of Digestive Cancer Research The Seventh Affiliated Hospital of Sun Yat‐sen University Shenzhen Guangdong 518107 China; ^2^ Department of Radiotherapy Affiliated Cancer Hospital of Zhengzhou University Henan Cancer Hospital Zhengzhou Henan 450000 China; ^3^ Department of Medicine The University of Oklahoma Health Sciences Center Oklahoma City OK 73104 USA; ^4^ Department of Gynecology and Obstetrics The Seventh Affiliated Hospital of Sun Yat‐sen University Shenzhen Guangdong 518107 China; ^5^ Department of Gastrointestinal Surgery Sun Yat‐sen Memorial Hospital Sun Yat‐sen University Guangzhou Guangdong 510120 China; ^6^ Department of Gastrointestinal Surgery The First Affiliated Hospital of Sun Yat‐sen University Guangzhou Guangdong 510062 China

**Keywords:** DEPTOR, gastric cancer, glycolysis, PI3K–Akt pathway, PUM1

## Abstract

RNA‐binding proteins (RBPs) play essential roles in tumorigenesis and progression, but their functions in gastric cancer (GC) remain largely elusive. Here, it is reported that Pumilio 1 (PUM1), an RBP, induces metabolic reprogramming through post‐transcriptional regulation of DEP domain‐containing mammalian target of rapamycin (mTOR)‐interacting protein (DEPTOR) in GC. In clinical samples, elevated expression of PUM1 is associated with recurrence, metastasis, and poor survival. In vitro and in vivo experiments demonstrate that knockdown of PUM1 inhibits the proliferation and metastasis of GC cells. In addition, RNA‐sequencing and bioinformatics analyses show that PUM1 is enriched in the glycolysis gene signature. Metabolomics studies confirm that PUM1 deficiency suppresses glycolytic metabolism. Mechanistically, PUM1 binds directly to DEPTOR mRNA pumilio response element to maintain the stability of the transcript and prevent DEPTOR degradation through post‐transcriptional pathway. PUM1‐mediated DEPTOR upregulation inhibits mTORC1 and alleviates the inhibitory feedback signal transmitted from mTORC1 to PI3K under normal conditions, thus activating the PI3K–Akt signal and glycolysis continuously. Collectively, these results reveal the critical epigenetic role of PUM1 in modulating DEPTOR‐dependent GC progression. These conclusions support further clinical investigation of PUM1 inhibitors as a metabolic‐targeting treatment strategy for GC.

## Graphical abstract

Pumilio 1 (PUM1), an RNA binding protein, induces metabolic reprogramming through post‐transcriptional regulation of DEP domain‐containing mammalian TOR (mTOR)‐interacting protein (DEPTOR) in gastric cancer (GC). This study reveals the critical epigenetic role of PUM1 in modulating DEPTOR‐dependent GC glycolytic metabolism, supporting further clinical investigation of PUM1 inhibitors as a metabolic‐targeting strategy for GC.

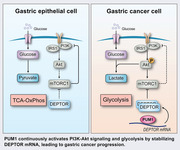

## 1 Introduction

Although the incidence and death rate of gastric cancer (GC) has decreased in recent years, it remains the fourth leading cause of cancer death worldwide.^[^ ^1^ ^]^ Multiple treatment methods including radical surgery, chemotherapy, targeted therapy, and immunotherapy have prolonged the survival and decreased the recurrence rate of GC.^[^ ^2^, ^3^, ^4^, ^5^ ^]^ However, the overall therapeutic effect on GC is still unsatisfactory. An in‐depth understanding of the intrinsic characteristic of GC is urgently necessary. Thus, it is important to explore the key pathogenic molecules and related mechanisms in the occurrence and progression of GC, which could be used to develop novel targeted drugs and improve survival rates.

RNA‐binding proteins (RBPs), which could bind directly to RNA, participate in post‐transcriptional regulation. RBPs forms ribonucleoprotein complex (RNP) with RNA and regulates the expression and function of target RNA (e.g., cutting pre‐mRNA, maintaining mRNA stability, and translation of mRNA).^[^ ^6^, ^7^, ^8^ ^]^ Abnormal regulation of RBPs is involved in tumorigenesis and progression; however, their functions and mechanisms remain largely elusive.^[^ ^9^, ^10^ ^]^ Pumilio 1 (PUM1), one type of sequence‐specific RNA‐binding protein, forms part of the eukaryotic Pumilio‐Fem3‐binding factor (PUF).^[^ ^11^, ^12^ ^]^ PUM1 contains a C‐terminal PUM homology domain, which consists of eight tandem helical repeats. This domain could form a crescent‐shaped superhelical structure with a concave surface, therefore, regulate various mRNAs via bind to specific single‐stranded RNA sequences.^[^ ^13^ ^]^ The precise role of PUM1 in tumor is controversial. It was recently reported to have housekeeping functions in certain tissues,^[^ ^14^, ^15^ ^]^ while other studies observed that it regulated the expression of tumor‐associated genes and was associated with tumor formation and growth. For instance, PUM1 promoted the growth and inhibited the apoptosis of myeloid leukemia cells by regulating the expression of FOXP1.^[^ ^16^ ^]^ In non‐small cell lung cancer, PUM1 promoted tumor proliferation via negatively regulated p27.^[^ ^17^ ^]^ However, no study has demonstrated the role of PUM1 in GC. Besides, the in‐depth mechanism of PUM1 in regulating carcinogenesis has not yet been fully explained.

In this study, we found an abnormally high expression of PUM1 in GC tissue, and this was connected with the patient's survival. In vitro and in vivo experiments provided further evidence that PUM1 could regulate tumor proliferation, metastasis, and glycolytic metabolism. Mechanistic studies showed that PUM1 could bind to the mRNA of DEPTOR and further stabilize its expression. Increased DEPTOR expression inhibited mTORC1 and alleviated the inhibitory feedback signal from mTORC1 to PI3K under normal conditions, resulting in a continuously activated PI3K–Akt signal that promoted the glycolytic metabolism in tumor cells. Overall, we elucidated a novel mechanism through which DEPTOR feedback activates the PI3K–Akt signal to change the metabolism of tumor cells. Our results revealed that PUM1 is essential for GC progression and these results may provide a possible targeted mechanism and strategy for treating GC in future.

## 2 Results

### 2.1 PUM1 Is Upregulated in GC and Can Predict Poor Prognosis

**Figure 1:**
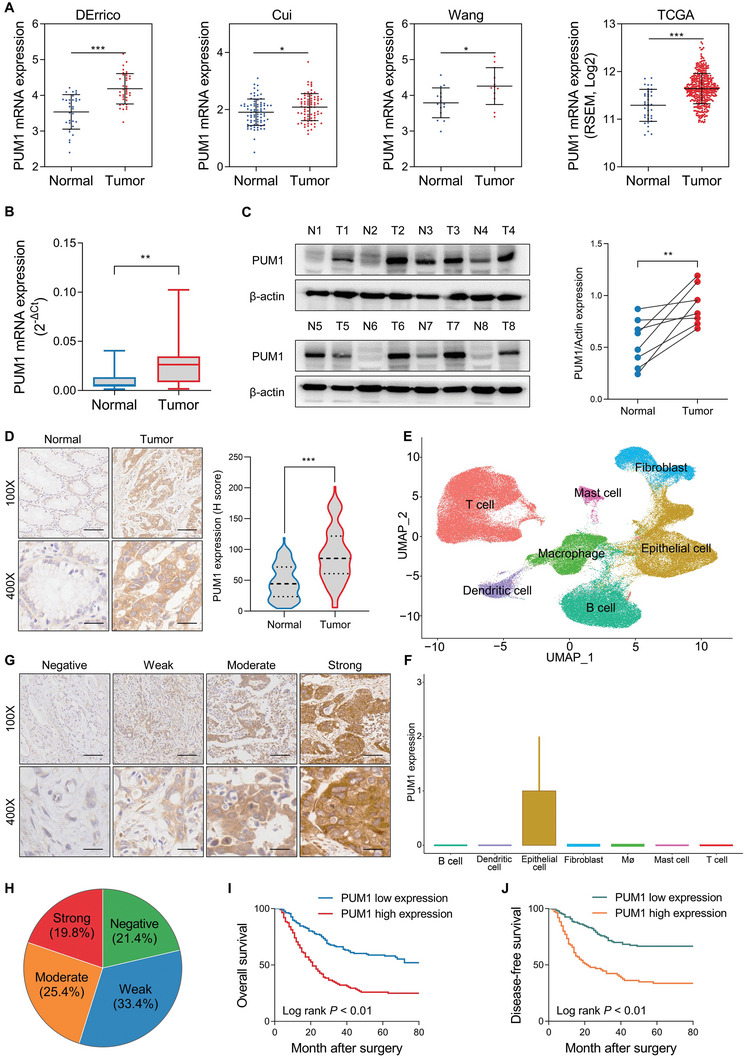
PUM1 is upregulated in GC and predicts poor prognosis. A) Expression difference of PUM1 between GC and normal tissues in the Oncomine platform and TCGA database. B) The mRNA expression levels of PUM1 in GC tissues and corresponding normal gastric tissues were detected by qRT‐PCR (*n* = 22). C) PUM1 protein levels in fresh GC and adjacent normal tissues detection by western blot (*n* = 8). D) Representative IHC images of PUM1 in GC tissue (*n* = 120) and corresponding normal tissue (*n* = 120). Scale bar, 100 µm. The corresponding IHC scores (*H*‐score) was compared on the right. E) Uniform manifold approximation and projection (UMAP) plots showed seven different cell types from GC single‐cell sequencing data GSE183904. F) PUM1 expression levels in different cell types were obtained from single‐cell sequencing data. G) Representative IHC images of PUM1 expression negative, weakly positive, moderately positive, and strongly positive in GC patients (*n* = 248). H) Proportion of different expression levels of PUM1 in GC patients. I,J) Kaplan–Meier analysis of overall survival (OS) or disease‐free survival (DFS) curves after having assigned GC patients to high/low of PUM1 expression subgroups. ^*^, *P* < 0.05; ^**^, *P* < 0.01; and ^***^, *P* < 0.001.

Oncomine platform and the Cancer Genome Atlas (TCGA) database were used to analyze the expression of PUM1 expression. The data from four independent cohorts consistently showed that GC tissues had significantly higher mRNA levels than normal gastric ones (**Figure** **1**A). To further identify this result, quantitative real‐time polymerase chain reaction (qRT‐PCR) (Figure 1B) and western blot (Figure 1C) detection was performed on fresh GC, and adjacent normal tissues of patients underwent gastrectomy in our center. Furthermore, immunohistochemistry (IHC) staining was performed on tissue sections from a cohort containing 120 GC patients (Figure 1D). These results revealed that compared with normal gastric tissues, GC exhibited significantly higher PUM1 expression. Moreover, we detected the expression of PUM1 in the primary and metastatic lesions of GC and found that PUM1 expression was higher in the metastatic lesions (Figure S1, Supporting Information). A public single‐cell sequencing data (GSE183904) was also analyzed. All cells were assembled and defined into seven cell types: B cell, T cell, dendritic cell, epithelial cell, fibroblast, macrophage, and mast cell (Figure 1E). The annotation cell markers were depicted in Figure S2 (Supporting Information). Among the seven cell types, PUM1 was mainly expressed in epithelial cell, which was mainly composed of tumor cells (Figure 1F). This result indicated that PUM1 was highly expressed on tumor cells in GC.

To demonstrate the clinical significance of PUM1 in GC, IHC detection was carried out using paraffin sections of GC tissues from 248 patients with radical gastrectomy. The results demonstrated that 45.2% of the patients displayed an aberrant expression of PUM1 (Figure 1G,H). Moreover, increased expression of PUM1 was also associated with tumor size, pathologic TNM (pTNM) stage, lymph node metastasis, lymphatic vessel invasion, and depth of invasion (Table S1, Supporting Information). In addition, patients with higher expression of PUM1 had worse disease‐free survival (DFS) as well as overall survival (OS) (Figure 1I,J). Finally, the increased expression of PUM1 was independent prognostic risk factor for GC (Table S2, Supporting Information).

### 2.2 PUM1 Deficiency Inhibits GC Proliferation In Vitro and In Vivo

**Figure 2:**
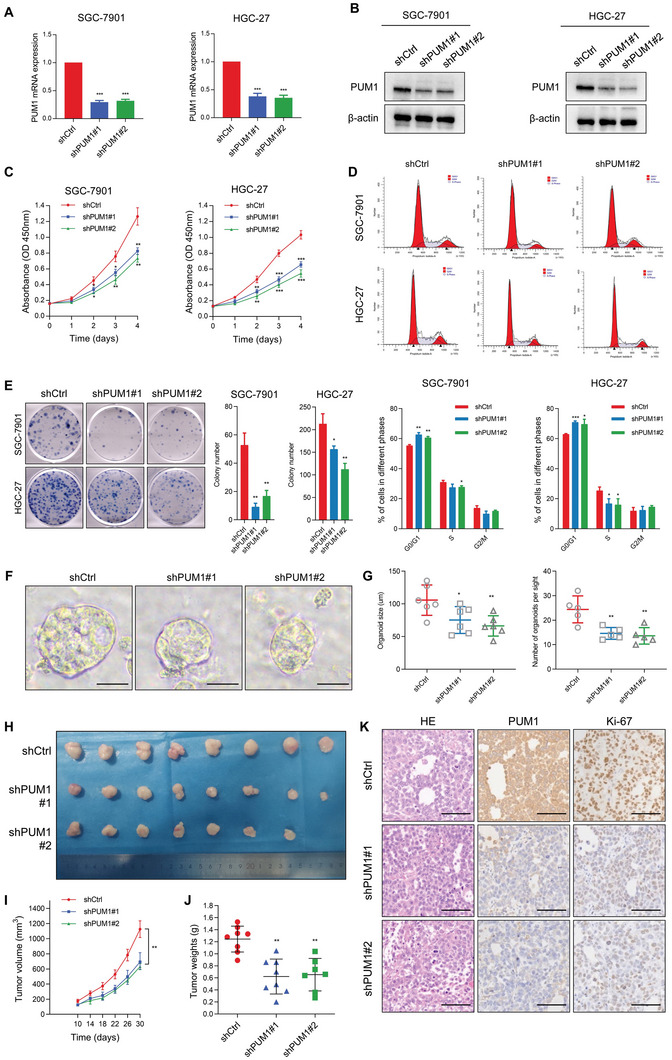
PUM1 deficiency inhibits GC proliferation in vitro and in vivo. A,B) Quantitative RT‐PCR and western blotting for PUM1 expression indicated in GC cell lines with stable PUM1 knockdown. C) Cell proliferation after knocking down PUM1 in SGC‐7901 and HGC‐27 cells determined by CCK‐8. D) Flow cytometric analysis of cell cycle on the 7th day after transfection of shPUM1s. E) Representative pictures of tumor plate cloning and statistics of colony counts of indicated cells. F) Representative image of organoid formation assay after knocking down PUM1. Scale bar, 50 µm. G) Statistics of diameter and number of organoids. H) Photograph of dissected subcutaneous xenografts (*n* = 8 per group). I) Growth curve of tumors in mice after subcutaneous xenografting using the indicated stable cell lines. J) Comparison of tumor weight of each group at last time point. K) Representative HE staining and IHC results of PUM1 and Ki‐67 in xenografted tumors. Scale bar, 100 µm. Data represent mean ± SD of three independent experiments. Error bars indicate mean ± SD. ^*^, *P* < 0.05; ^**^, *P* < 0.01; and ^***^, *P* < 0.001.

PUM1 was highly expressed in HGC‐27 and SGC‐7901 GC cell lines (Figure S3, Supporting Information). We constructed stable knockdown PUM1 cells in these two cell lines using short hairpin RNA (shRNA). **Figure** **2**A,B shows the transfection efficiency as detected by qRT‐PCR and western blotting, respectively. Cell cycle, plate colony formation, and CCK8 cell proliferation assays were performed to assess the proliferation ability of PUM1 in vitro. As the results showed, deficiency of PUM1 significantly reduced the cell viability (Figure 2C), and the number of cell colonies (Figure 2E). Deficiency of PUM1 induced the accumulation in the G0/G1 phase (Figure 2D). Moreover, organoid‐formation assay showed that silencing PUM1 caused a decrease in the size and number of human GC organoids (Figure 2F).

Furthermore, subcutaneous injections of PUM1‐deficient SGC‐7901 cells in BALB/c nude mice were used to determine the tumorigenesis in vivo. Consistently, PUM1 deficiency slowed down the proliferation of SGC‐7901 engrafted tumors (Figure 2H,I), with those in which PUM1 was knocked down also having a significantly lower weight than PUM1 competent tumors (Figure 2J). IHC staining of the tumor sections showed that knockdown of PUM1 significantly reduced the expression of Ki67 (Figure 2K), indicated reduced proliferation ability. Taken together, the above results indicated that PUM1 could promote tumorigenesis in vitro and in vivo.

### 2.3 PUM1 Is Required for Tumor Metastasis, Invasion, and Peritoneal Dissemination Of GC

**Figure 3:**
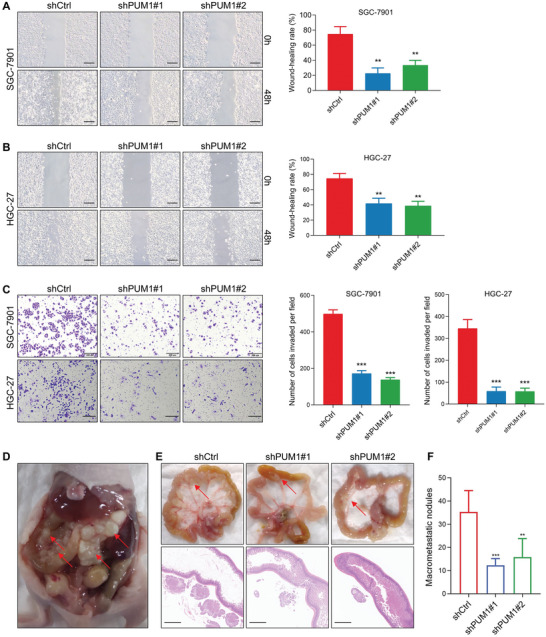
PUM1 is required for tumor metastasis, invasion, and peritoneal dissemination of GC. A,B) Microscopic images and quantification of indicated cells in the wound‐healing migration assays. Scale bar, 100 µm. C) Microscopic images and quantification of the invasiveness of the indicated cells in the Transwell matrix penetration assays. Scale bar, 200 µm. D) SGC‐7901 cells expressing control or PUM1 shRNAs were injected intraperitoneally and metastatic nodules in the colonic wall were recorded 6 weeks later. E) Representative macroscopic and microscopic HE staining images of mesenteric metastatic nodules (arrows). Scale bar, 1 mm. F) Statistical analysis of macroscopic metastatic nodules (*n* = 7 per group). Error bars indicate means ± SD. ^**^, *P* < 0.01; ^***^ and *P* < 0.001.

We further explored the effect of PUM1 on the migration and invasion of GC. The wound‐healing assay indicated that knocking down PUM1 significantly suppressed migration ability (**Figure** **3**A,B). Subsequently, the transwell invasion experiment showed that PUM1 knockdown could also reduce invasion ability (Figure 3C). Since peritoneal metastasis is the most common metastasis type in GC,^[^ ^18^ ^]^ we used peritoneal metastasis model to detect the effects of PUM1 on GC cell metastasis as our previous study described.^[^ ^19^ ^]^ The control cells and PUM1 knockdown cells were injected into peritoneal cavity, respectively. After 6 weeks, the mice were sacrificed and we found that metastases lesions were mostly detected in the mesentery. The results showed that knockdown of PUM1 significantly reduced the number of macro‐metastatic nodules, as demonstrated by hematoxylin‐eosin (HE) staining (Figure 3D–F). Altogether, these data proved that PUM1 was critical for tumor invasion, metastasis, and peritoneal dissemination of GC.

### 2.4 PUM1 Positively Regulates Glycolysis in GC

**Figure 4:**
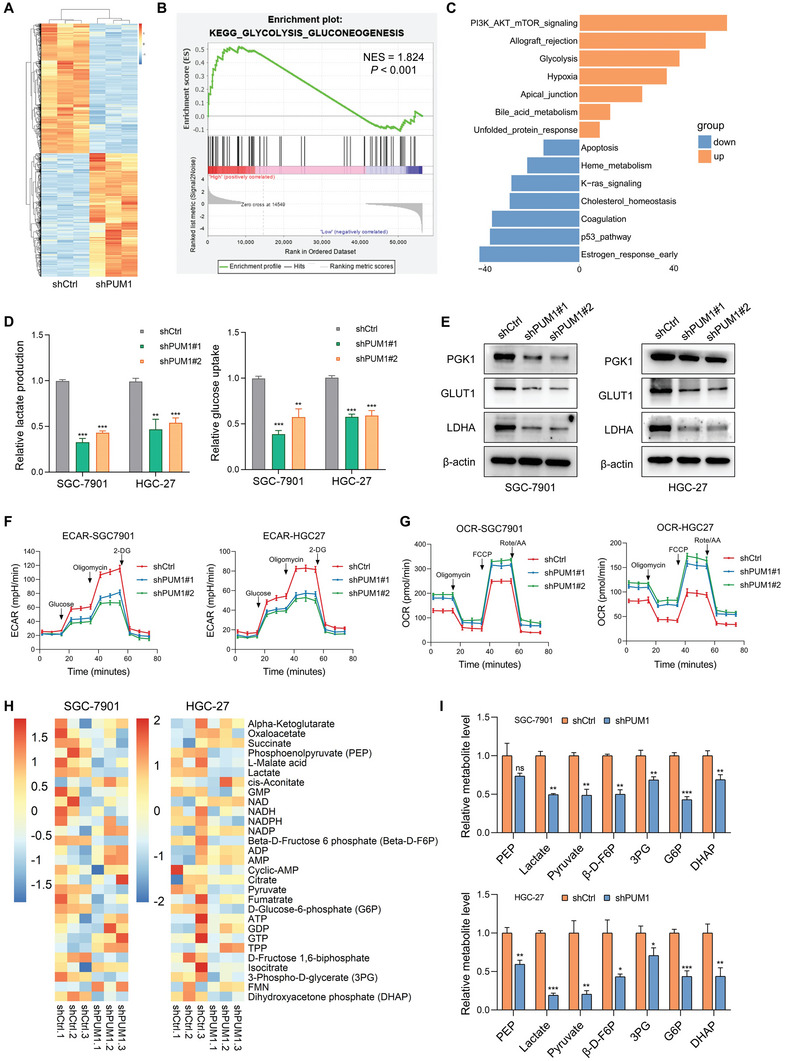
PUM1 positively regulates glycolysis in GC. A) Heatmap of all differential genes in SGC‐7901 cells that stably express shRNAs targeting PUM1 or scramble control. B) GSEA analysis showed that differentially expressed genes were mainly enriched in glycolysis pathway. C) GSVA analysis between PUM1‐high and low groups. D) Relative lactate production and glucose uptake of indicated cells transfected with PUM1 shRNAs or scramble control. E) Western blotting shows changes in the expression levels of glycolytic proteins PGK1, GLUT1, and LDHA by PUM1 knockdown or control cells. F,G) ECAR and OCR of indicated cells transfected with PUM1 shRNAs or scramble control were measured with seahorse. H) Heatmap of differential metabolites in PUM1 deficiency and control GC cells. I) Major metabolites altered in the glycolytic pathway in PUM1 deficiency and control GC cells. Error bars indicate means ± SD. ^*^, *P* < 0.05; ^**^, *P* < 0.01; and ^***^, *P* < 0.001.

In order to comprehensively understand the role of PUM1 in promoting GC progression, we performed RNA‐seq analyses with PUM1 knockdown and control SGC‐7901 cells. The RNA profiles showed that PUM1 deficiency was associated with the dysregulation of different subsets of transcripts (**Figure** **4**A). Gene set enrichment analysis (GSEA) indicated that differentially expressed genes were mainly enriched in glycolysis pathway (Figure 4B). Additionally, to explore different pathway enrichment between PUM1‐high and PUM1‐low cells, we conducted Gene Set Variation Analysis (GSVA) in single‐cell sequencing data. Higher PUM1 expression was enriched in glycolysis and PI3K–Akt pathway (Figure 4C). Glycolysis is one of the primary glucose metabolic signatures in cancer. In contrast to normal cells, tumor cells could produce energy by uptaking more glucose and glycolysis, leading to lactic acid fermentation. This process is termed aerobic glycolysis, the so‐called Warburg effect, is an important characteristic of actively proliferating tumor cells.^[^ ^20^, ^21^ ^]^ Hence, we hypothesized that PUM1 may influence the proliferation and metastasis of GC cells via regulating the glycolysis. To validate this hypothesis, the glucose and lactic acid content in culture medium of GC cells were measured. The results showed that PUM1 knockdown could reduce glucose absorption and lactic acid production (Figure 4D). With the Seahorse XF Extracellular Flux Analyzers, measurement of extracellular acidification rate (ECAR) and oxygen consumption rate (OCR) was used to determine the aerobic glycolysis of GC. The results demonstrated that PUM1 deficiency significantly decreased the ECAR value (Figure 4F) and increased the OCR value in GC cells (Figure 4G). To obtain more comprehensive insight into this phenotype, we detected the expression of rate‐limiting enzymes involved in glycolysis. Proteins such as lactate dehydrogenase A (LDHA), phosphoglycerate kinase 1 (PGK1), and glucose transporter 1 (GLUT1)^[^ ^22^ ^]^ are required for glucose uptake as well as for the conversion of pyruvate to lactate, respectively. Nonetheless, PUM1‐deficient cells had significantly lower expression of these proteins (Figure 4E). Consistently, analysis from TCGA database also suggested that PUM1 expressions was positively correlated with glycolysis‐related gene, such as GLUT1 (SLC2A1), HK2, PGK1, and GPI (Figure S4, Supporting Information). Further LC/MS targeting metabolomics analyses demonstrated that PUM1 deficiency induced decrease of glucose 6‐phosphate (G6P), 3‐phospho‐d‐glycerate (3PG), pyruvate, dihydroxyacetone phosphate (DHAP), and lactate, which were metabolites in glycolysis pathway (Figure 4H,I). Altogether, the results suggested that PUM1 was a positive regulator of glycolysis.

### 2.5 DEPTOR Is a Binding and Regulation Target of PUM1

**Figure 5:**
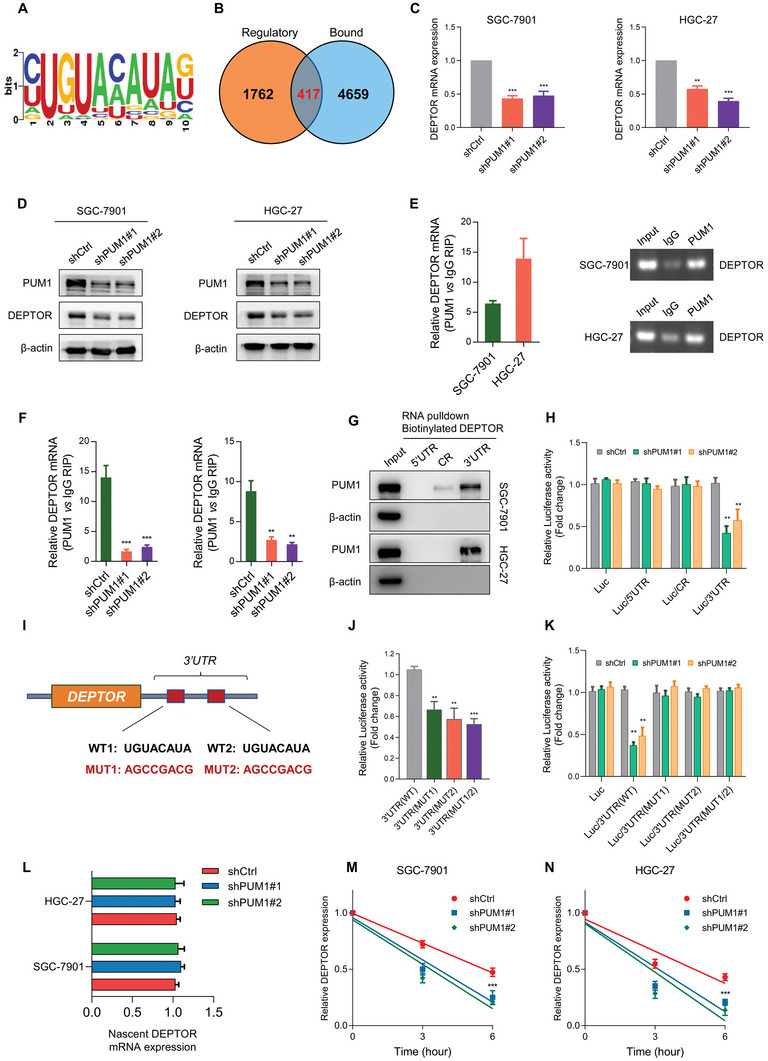
DEPTOR is a binding and regulation target of PUM1. A) Sequence logo for PUM1 binding motif. B) Differential expressed genes (DEGs) in RNA sequencing data were defined as potential regulatory group. Genes containing a PUM1‐binding motif were defined as the potential binding group. Overlap between the “Regulatory” and “Bound” datasets was shown. C) Quantitative RT‐PCR analysis showing changes in RNA levels of DEPTOR expression in indicated GC cell lines with PUM1 knockdown or scramble control. D) Western blotting showing changes in protein levels of DEPTOR expression by PUM1 knockdown or control cells. E) RIP was carried out with cell lysates using PUM1 antibody, with RT‐PCR (left) and agarose electrophoresis (right) used to determine DEPTOR mRNA enrichment. F) RIP–qRT‐PCR shows enriched DEPTOR in indicated cells after inhibiting PUM1. G) Biotinylated RNA segments of DEPTOR mRNA (5′UTR, CR, and 3′UTR) were used to pull down lysates from indicated cells. RNA pulldown materials were detected by western blotting, with the use of PUM1 antibody. H) Relative luciferase activity was detected and normalized by Renilla activity in indicated groups of SGC‐7901 cells. I) Schematic diagram of PUM1‐binding motifs (wild‐type, WT) and corresponding mutations (MUT) on DEPTOR mRNA 3′UTR. J) Relative luciferase activity was analyzed in SGC‐7901 cells transfected with wild‐type or mutant DEPTOR 3′UTR luciferase reporter vector. K) Relative luciferase activity was analyzed in PUM1 knockdown or control SGC‐7901 cells transfected with wild‐type or mutant DEPTOR 3′UTR luciferase reporter vector. L) Nascent synthesized DEPTOR mRNA was labeled and detected by qRT‐PCR in indicated cells. M,N) Actinomycin D (4 µg mL^−1^) was used to treat PUM1 knockdown or control SGC‐7901 and HGC‐27 cells. The attenuation of DEPTOR mRNA was detected by qRT‐PCR at 0, 3, and 6 h after actinomycin D treatment. Error bars indicate means ± SD. ^**^, *P* < 0.01 and ^***^, *P* < 0.001.

As a highly conserved RNA‐binding protein, PUM1 exerts biological functions by binding to target mRNA and affecting post‐transcriptional regulation.^[^ ^11^, ^12^, ^23^ ^]^ PUM1 binds with high affinity and specificity to the target motif referred to as Pumilio Response Element (PRE) with the consensus 5′‐UGUANAUA‐3′ (where N is A, C, G, or U)^[^ ^23^, ^24^ ^]^ (**Figure** **5**A). We intersected the set of genes containing at least one PRE with differentially expressed genes in transcriptome sequencing. Finally, we obtained 417 candidate genes, which may be potential direct PUM targets (Figure 5B). Among these candidate genes, DEP domain‐containing mammalian TOR (mTOR)‐interacting proteins (DEPTOR) displayed differential expression in transcriptome sequencing, which could participate in glucose homeostasis and play an important role in metabolic signaling pathways.^[^ ^25^ ^]^ Therefore, we hypothesized that PUM1 could directly bind with the mRNA of DEPTOR and regulate the expression. To test this hypothesis, mRNA and protein of DEPTOR were examined, respectively. After knocking down PUM1, the expression of DEPTOR significantly decreased in both SGC‐7901 and HGC‐27 cells (Figure 5C,D). RNA immunoprecipitation (RIP) assay revealed that PUM1 could enrich DEPTOR mRNA compared with IgG (Figure 5E), while PUM1 deficiency reduced the enrichment (Figure 5F). Furthermore, RNA pulldown assay was carried out using the transcribed 3′Untranslated region (UTR), CR or 5′UTR part of the DEPTOR mRNA. The results showed that PUM1 successfully pulled down only by the 3′UTR of DEPTOR (Figure 5G). Consistent with the RNA pulldown assay, the luciferase activity of SGC‐7901 cells with PUM1 deficiency was significantly lower, especially for constructs containing the 3′UTR of DEPTOR (Figure 5H). Then, we screened and identified two potential PREs on the DEPTOR mRNA sequence. We further constructed luciferase constructs containing DEPTOR 3′UTR with PRE mutations (MUT, Figure 5I). Luciferase analyses confirmed that each of the two MUT PREs decreased luciferase activity (Figure 5J). Additionally, PUM1 knockdown only reduced the luciferase activity of the wild‐type (WT) 3′UTR fragment without changing two MUT 3′UTR fragments (Figure 5K). These data indicated that PUM1 could bind with the 3′UTR of DEPTOR mRNA and promote its expression.

Next, we tested whether PUM1 affected the production of DEPTOR mRNA by labeling newly synthesized RNA with the Click‐iT nascent RNA capture system. There were no clear changes in the newly synthesized DEPTOR RNA in cells with PUM1 silence (Figure 5L). In addition to RNA production, RNA degradation may also affect DEPTOR expression. Actinomycin D was used to block de novo RNA synthesis, and the persistence of existing DEPTOR mRNA was measured by qRT‐PCR. Compared with control cells, PUM1 knockdown promoted degradation of the DEPTOR mRNA in GC cells (Figure 5M,N). Altogether, these results demonstrated that PUM1 could directly recognize the PREs on DEPTOR mRNA and maintain transcript stability, thereby preventing their degradation and increasing the expression level.

### 2.6 PUM1 Regulates PI3K–Akt Signaling Pathway and Glycolysis through DEPTOR

**Figure 6:**
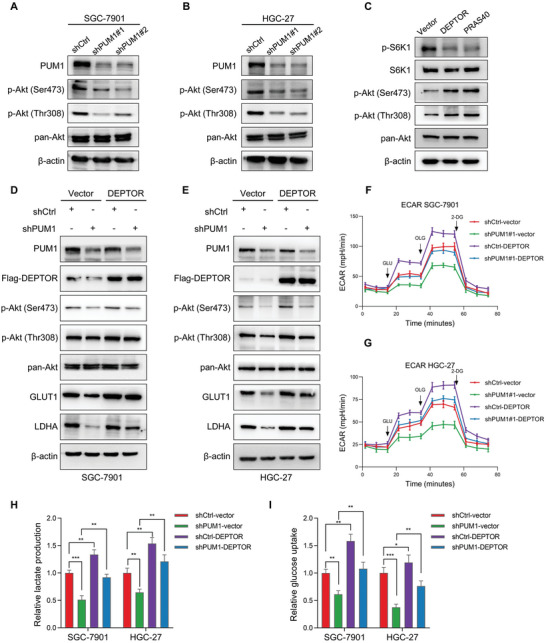
PUM1 regulates PI3K–Akt signaling pathway and glycolysis through DEPTOR. A,B) Western blot detected the changes in pan‐Akt and phosphorylated Akt (p‐Akt) levels in SGC‐7901 and HGC‐27 cells with PUM1 knockdown or scramble control. C) DEPTOR or PRAS40 was overexpressed in SGC‐7901 cells and protein levels of S6K1, phosphorylated S6K1, phosphorylated Akt, and pan‐Akt were detected by western blotting. D,E) Western blotting of p‐Akt, pan‐Akt, GLUT1, and LDHA in PUM1 knockdown SGC‐7901 and HGC‐27 cells after transfection with DEPTOR constructs or empty vector control. F,G) ECAR values of SGC‐7901 and HGC‐27 cells as described above were detected by seahorse. H,I) Relative lactate production and glucose uptake of SGC‐7901 and HGC‐27 cells as described above. Error bars indicate means ± SD. ^*^, *P* < 0.05; ^**^, *P* < 0.01; and ^***^, *P* < 0.001.

DEPTOR acted as a negative regulator of mTOR by binding to mTORC1 and mTORC2.^[^ ^25^ ^]^ We found that phosphorylated Akt (p‐Akt) significantly decreased in PUM1 deficient cells, while there was little change in total Akt protein (**Figure** **6**A,B). In addition, overexpression of DEPTOR or PRAS40 (a widely recognized mTORC1 inhibitor) in SGC‐7901 cells decreased the phosphorylation of S6K1 (an mTORC1 substrate) (Figure 6C), which suggested that mTORC1 was inhibited. mTOR was not only a downstream effector of the PI3K–Akt signal, but also a feedback regulator of Akt activity.^[^ ^25^, ^26^ ^]^ We assumed that overexpressed DEPTOR inhibited mTORC1 and alleviated the inhibitory feedback signal transmitted from mTORC1 to PI3K under normal conditions. This may induce continuous activation of the PI3K signal, which is in line with the previous study.^[^ ^26^ ^]^

The activation of PI3K–Akt pathway could promote glycolytic metabolism in cancer cells.^[^ ^21^, ^27^ ^]^ Given the observed regulatory effect of DEPTOR on Akt activity, we hypothesized that PUM1 could regulate the PI3K–Akt pathway and glycolysis by interacting with DEPTOR. We found that increased activation or expression of Akt phosphorylation, LDHA, and GLUT1 when DEPTOR was overexpressed in PUM1 deficient GC cells (Figure 6D,E). Further analyses revealed that partial recovery in ECAR, lactate production, and glucose uptake were observed when DEPTOR was overexpressed in PUM1 knockdown cells (Figure 6F–I). Next, we investigated whether PUM1–DEPTOR‐regulated glycolysis was dependent on Akt signaling. Upregulation of LDHA and GLUT1 caused by DEPTOR overexpression was reversed by adding the Akt inhibitor MK‐2206 (10 µm, 24 h) (Figure S5, Supporting Information). These results indicated that PUM1 activated PI3K–Akt signaling via regulating DEPTOR, thereby affecting glycolysis in GC.

### 2.7 The PUM1–DEPTOR–Akt Axis Promotes GC Progression and Is a Potential Therapeutic Target

**Figure 7:**
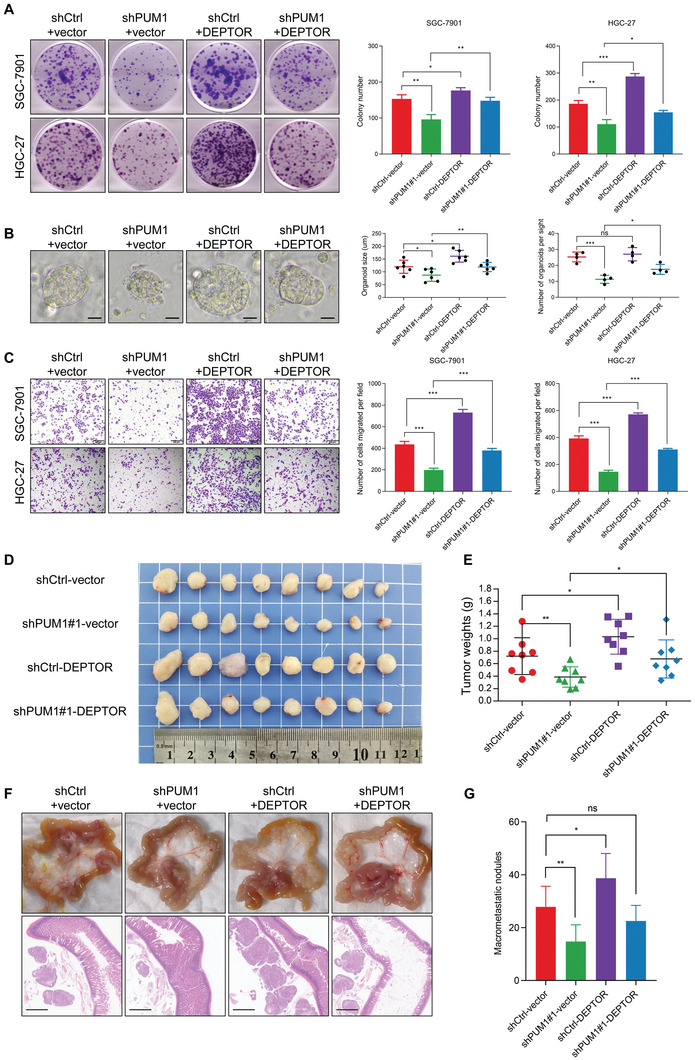
PUM1–DEPTOR–Akt axis contributes to GC progression. A) Representative images of clone formation and statistics of colony counts in PUM1 knockdown SGC‐7901 and HGC‐27 cells after transfection with DEPTOR constructs or empty vector. B) Representative image and statistics of diameter and number of organoids. Scale bar, 50 µm. C) Microscopic images and quantification of the invasiveness of SGC‐7901 and HGC‐27 cells as described above. D) Subcutaneous xenografted SGC‐7901 cells expressing PUM1 shRNAs and/or DEPTOR constructs. The image of dissected subcutaneous xenografts (*n* = 8 per group). E) Comparison of tumor weight of each group at last time point. F) Intraperitoneal injected SGC‐7901 cells expressing PUM1 shRNAs and/or DEPTOR constructs. Representative macroscopic and microscopic HE staining images of mesenteric metastatic nodules. Scale bar, 1 mm. G) Statistical analysis of macroscopic metastatic nodules (*n* = 7 per group). Error bars indicate means ± SD. ^**^, *P* < 0.01 and ^***^, *P* < 0.001.

**Figure 8:**
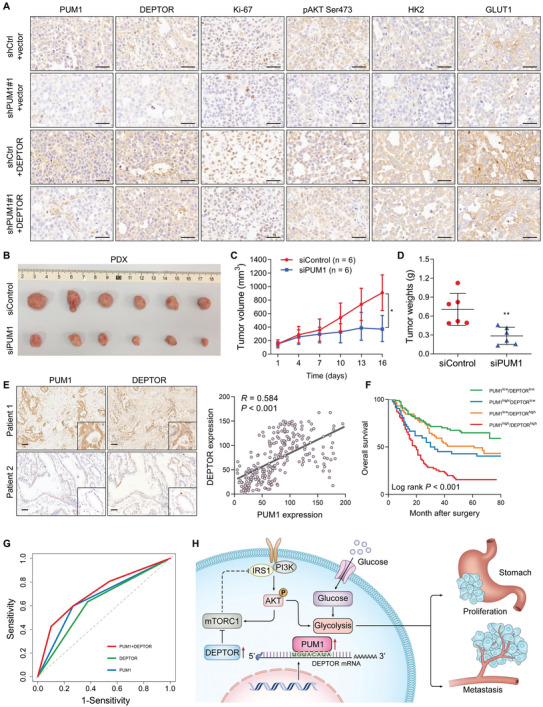
Clinical value of PUM1–DEPTOR–Akt axis in GC. A) Representative IHC staining of PUM1, DEPTOR, Ki‐67, p‐Akt, HK2, and GLUT1 in xenografted tumors. Scale bar, 50 µm. B) Photograph of the excised tumors from PDX model after intratumoral injection of siPUM1 or the control (*n* = 6 per group). C) The tumor growth analysis of PDX model with siControl or siPUM1 treatment. D) Comparison of tumor weight at last time point with siControl or siPUM1 treatment. E) Representative images of PUM1 and DEPTOR IHC staining in 248 GC patient specimens. Scale bar, 100 µm. Right: correlational analyses highlighted a significant link between PUM1 and DEPTOR expression in patient specimens (Pearson correlation, *R* = 0.584). F) Kaplan–Meier analysis of overall survival of PUM1 and DEPTOR combinations in our cohort. G) Receiver operating characteristic (ROC) curve analysis of PUM1 (area under a curve AUC = 0.665) or DEPTOR (AUC = 0.628) single scoring or combinational scoring (AUC = 0.713). H) Schematic diagram of PUM1 regulating GC progression via DEPTOR.

We further explored the effects of DEPTOR on PUM1‐mediated proliferation and metastasis of GC. We overexpressed DEPTOR in PUM1 knockdown and control GC cells. In vitro experiments showed that, compared with an empty vector, overexpressing DEPTOR could enhance the clonogenic, organoid formation, and invasive ability of GC cells (**Figure** **7**A–C). Upregulated DEPTOR in PUM1 deficiency cells could restore certain clonal tumorigenesis and invasion ability. The subcutaneous tumor transplantation experiment and abdominal metastasis experiment in mice further confirmed that overexpression of DEPTOR after silencing PUM1 could restore the proliferation and metastasis of cells in vivo (Figure 7D–G). IHC results of transplanted tumors in mice showed that the DEPTOR, p‐Akt, HK2, GLUT1, and Ki‐67 were downregulated in PUM1‐deficient tumors (**Figure** **8**A). Conversely, these proteins were upregulated in tumors with DEPTOR overexpression (Figure 8A).

The patient‐derived xenograft (PDX) model closely resembles the biological characteristics and genomic landscape of human cancers at the population level.^[^ ^28^, ^29^ ^]^ To further confirm whether PUM1 could be used as a therapeutic target in GC, we assessed the antitumor activity of PUM1‐targeting siRNA in the PDX model. When the tumor volume reached ≈150 mm^3^, siRNA targeting PUM1 or siControl was injected intratumorally every 3 days. Our results showed that tumor growth and weight were significantly suppressed after treatment with siPUM1 (Figure 8B–D). In addition, we evaluated the effect of PUM1 depletion on normal gastric tissue using the organoid model. We found that knocking down PUM1 did not significantly affect the growth of normal gastric organs (Figure S6, Supporting Information). This might be because normal cells had low levels of PUM1 expression and glycolysis. Our study confirms PUM1 as a valuable therapeutic target for GC and provides preclinical exploration.

To investigate the clinical correlation between PUM1 and DEPTOR, IHC was used to assess the expression in GC specimens. The results showed that PUM1 staining was positively correlated with DEPTOR levels (Figure 8E). Based on the PUM1 and DEPTOR expressions, GC patients were categorized into four groups with different expressions (Figure 8F). Patients with both PUM1 and DEPTOR being highly expressed had the worst survival. Moreover, combination of PUM1 and DEPTOR immunostaining showed a better predictive performance of survival in GC patients than either parameter alone (Figure 8G). Together, these data provided support for the model suggesting that PUM1 positively regulates DEPTOR in a clinical context as a tumor promoter.

## 3 Discussion

PUM1 is involved in the progression of some tumors;^[^ ^30^, ^31^, ^32^, ^33^ ^]^ however, the mechanism was not elucidated. In this study, we demonstrated that PUM1 directly binds to DEPTOR mRNA PRE to maintain the stability of the transcript and prevent DEPTOR degradation through post‐transcriptional pathway. PUM1‐mediated promotion of DEPTOR expression inhibited mTORC1, preventing the normal inhibitory feedback between mTORC1 and PI3K and leading to continuous activation of PI3K–Akt signaling and glycolysis (Figure 8H).

PUM1 binds specifically, along with high affinity, to the target motif of the PRE, with the recognition sequence 5′‐UGUANAUA‐3′.^[^ ^24^, ^34^ ^]^ We intersected gene sets containing at least one PRE and differentially expressed genes from transcriptome sequencing. Among the metabolism‐related differential genes, DEPTOR caught our attention because its 3′UTR includes PUM1 potentially binding PRE sequence. As an mTOR‐interacting protein containing the DEP structure domain, DEPTOR is important for cell metabolism signaling, including involvement in adipogenesis and glucose homeostasis. Previously, studies reported that DEPTOR regulates myocyte glycolysis in the isokinetic contraction of skeletal muscle.^[^ ^35^ ^]^ Higher levels of DEPTOR were found in the glycolytic muscle of mice with Baf60c overexpression. Our study confirmed that PUM1 positively regulates DEPTOR protein and RNA expression. RIP and luciferase assay clarified that PUM1 could target and bind the 3′UTR of DEPTOR mRNA. Hence, DEPTOR represents a newly discovered target gene directly regulated by PUM1. DEPTOR can promote or inhibit cancer in different types of tumors. Low DEPTOR levels have been reported in lung adenocarcinoma.^[^ ^36^ ^]^ In addition, low DEPTOR mRNA and protein levels correlate with a high EGFR signaling pathway. Still, in colon cancer, DEPTOR acts as a tumor promoter that is targeted by the Wnt/b‐Catenin/c‐Myc signaling pathway.^[^ ^37^ ^]^ Similarly, in T‐cell leukemia, the NOTCH1 signal is abnormally activated, promoting cell survival and proliferation through DEPTOR.^[^ ^38^ ^]^ Results of in vitro and in vivo experiments indicated that DEPTOR overexpression could promote proliferation, metastasis, and glycolysis of GC cells. In addition, overexpressing DEPTOR in PUM1‐silenced GC cells can partially restore the inhibition of cell proliferation and glycolysis caused by PUM1 deficiency. Our results revealed DEPTOR as a novel target gene of PUM1, which promotes proliferation, metastasis, and glycolytic metabolism through DEPTOR in GC.

We observed that PUM1 deficiency inhibits Akt phosphorylation in GC cells. DEPTOR acts as a negative regulator of mTOR by binding to both mTORC1 and mTORC2,^[^ ^25^ ^]^ which are significantly upregulated in the majority of human tumors. Conversely, DEPTOR was also reported to have cancer‐promoting functions in some tumors.^[^ ^37^, ^38^, ^39^, ^40^ ^]^ Indeed, mTOR was not only a downstream effector of the PI3K–Akt signal, but also a feedback regulator of Akt activity.^[^ ^25^, ^26^ ^]^ Previously, in vitro kinase assays demonstrated that DEPTOR consumption increased the activation of S6K1 by mTORC1 and Akt by mTORC2.^[^ ^26^ ^]^ However, in cell culture, it was found that the regulation of Akt by DEPTOR was not consistent with the determination of external kinase, which was more complex than expected.^[^ ^26^ ^]^ When overexpressing DEPTOR or PRAS40 in GC cells, lower phosphorylation of S6K1 was observed, indicating that mTORC1 was inhibited. Interestingly, DEPTOR overexpression increased Akt phosphorylation. We assumed that overexpressing DEPTOR inhibited mTORC1, thereby disengaging the inhibitory signal normally delivered by mTORC1 to PI3K. Similarly, overexpression of DEPTOR was also found to promote Akt activation in myeloma cells.^[^ ^26^ ^]^ Our results suggest that PUM1 positively regulates DEPTOR to activate the Akt signal. In transmitting signals from cell membrane receptors to the nucleus, PI3K/Akt carries out intracellular transduction of proliferative signals. In particular, activation of PI3K/Akt enhances glucose uptake and glycolysis.^[^ ^41^, ^42^ ^]^ This increases the expression of glucose transporters on the cell surface, activating hexokinase and capturing intracellular glucose via phosphorylation, leading to increased lactate conversion for glycolytic glucose metabolism and the promotion of cell growth, division, and metastasis.^[^ ^20^, ^43^, ^44^ ^]^ Our results showed that ECAR and glycolysis‐related proteins increased with DEPTOR upregulation and Akt activation in GC cells. In addition, DEPTOR overexpression could restore the glycolytic inhibition induced by silencing PUM1. These results revealed that PUM1 activated Akt through DEPTOR, thus promoting glycolysis in GC cells.

The most classical function of PUM1 is post‐transcriptional repression. mRNA expression is inhibited by PUF proteins through recruitment of the Ccr4–Pop2–NOT deadenylase complex or by blocking interactions between the 5′mRNA cap and translation initiation factors. This alters the structures of ribonucleoproteins and can also limit the extension and termination steps of the translation process.^[^ ^11^ ^]^ PUM1 can also induce local conformational changes to facilitate binding to microRNAs.^[^ ^32^, ^45^ ^]^ Activator function of PUM1 has been reported in African clawed toads and *Cryptobacterium hidradenum*, which are associated with polyA tail extension or polyA‐dependent translational activation.^[^ ^46^, ^47^ ^]^ We discovered that PUM1 binds to PRE in the DEPTOR–3′UTR and promotes DEPTOR expression. Labeling newly synthesized RNA by a nascent RNA capture system suggested that the PUM1‐promoted expression did not occur due to an increase in newly synthesized DEPTOR. Furthermore, we examined the effect of PUM1 on DEPTOR–mRNA degradation. Blocking RNA synthesis with actinomycin D resulted in a significantly faster rate of DEPTOR–mRNA decay in PUM1‐inhibited cells. Thus, we identified a new way for PUM1 to promote target gene expression, which was named by binding to DEPTOR mRNA, thus making it more stable in inhibiting degradation.

In summary, our results reveal a novel PUM1–DEPTOR–Akt axis that is critical to cause metabolic reprogramming and tumor progression in GC. These results may provide a novel prognostic indicator and promising therapeutic target for GC.

## 4 Experimental Section

### Patients and Tissue Samples

Primary GC tissues and adjacent nontumor tissues were collected from 248 GC patients who underwent radical gastrectomy at The First Affiliated Hospital, Sun Yat‐sen University, between 2009 and 2013. A total number of 24 GC metastatic tissues were also collected including liver metastases, ovarian metastases, and peritoneal metastases. For patients with radical gastrectomy, information regarding their clinicopathological characteristics and survival outcomes was collected, and none of them had received radiotherapy or chemotherapy prior to the surgery. In addition, the 7th Edition of American Joint Committee on Cancer (AJCC) TNM staging system was applied to categorize the stage of the tumors. PUM1 expression levels were quantified before being linked to the collected clinicopathological factors and prognosis data of patients. All patients were required to provide written informed consent for this study which was undertaken with the approval of the ethics committee of The First Affiliated Hospital, Sun Yat‐sen University (KY‐2021‐118‐01).

### ECAR and OCR Measurements

The OCR and the ECAR which reflect cellular mitochondrial respiration and glycolytic capacity, respectively, were determined with a Seahorse Bioscience XF96 Extracellular Flux Analyzer. In this case, the procedure specified in the Cell Mito Stress Test Kit and the Seahorse XF Glycolysis Stress Test Kit (Seahorse Bioscience) was followed.

### Transcriptome Sequencing

PUM1 control or knocked down SGC‐7901 cells were used for extracting total RNA, which were then used for RNA sequencing, with analysis subsequently performed by Seqhealth Technology Co., Ltd. (Wuhan, China). RNA sequencing data were deposited to the NCBI database under accession number GSE212848.

### Labeling and Capture of Nascent RNA

Click‐iT Nascent RNA Capture kit (Invitrogen) was used for detecting newborn RNA as described before.^[^ ^48^ ^]^ This involved incubating cells with 200 µm ethylene uridine reagent for 6 h. Then cells were lysed and the RNA was isolated. A biotin azide was “clicked on” and the newly synthesized pool of RNA was captured with streptavidin magnetic beads. The captured RNAs were then amplified and the resultant cDNAs were used on qRT‐PCR analysis.

### RNA Stability Assays

To measure the RNA stability of DEPTOR, cells were treated with 4 µg mL^−1^ actinomycin D at 0, 3, and 6 h. TRIzol reagent was then used for extracting total RNA prior to qRT‐PCR. For each treatment time, the mRNA expression levels of cells were determined and normalized to β‐actin.

### RIP Assay

RIP was performed with the Magna RBP immunoprecipitation kit (Millipore #17‐700) as specified by the manufacturer. This involved lysing 1 × 10^7^ cells with RIP lysis buffer before extracting the supernatant. Immunoprecipitation was then performed with 5 µg of PUM1 antibody while for a corresponding control rabbit IgG, protein A/G magnetic beads were used. Washing of magnetic bead–bound complexes was then followed by incubation with proteinase K. Input and co‐immunoprecipitation RNAs were inverse transcription and performed RT‐PCR.

### RNA Pulldown Assay

RNA was amplified with the MEGAscript T7 Transcription Kit (Thermo Scientific). In this case, DNA, which was amplified by PCR, provided the template for the synthesis of biotinylated RNA by T7 RNA polymerase and in the presence of biotin UTP. Using the Pierce Magnetic RNA‐Protein Pull‐Down Kit (Thermo Scientific), RNA pulldown was then performed. Finally, the streptavidin‐conjugated beads were boiled, and the pulldown materials were used for the immunoblotting analysis.

### Dual luciferase Reporter Assay

PCR amplification of full‐length 3′UTR, CR, and 5′UTR of DEPTOR was performed, with mutational 3′UTR fragments also created by site‐directed mutagenesis. These fragments were then cloned into a pMIR‐REPORT Luciferase vector (Promega). GC cells infected with lentiviruses containing scramble or shPUM1 were co‐transfected with both the above firefly luciferase reporter vector and a Renilla luciferase vector. Eventually, the Dual Luciferase Assay Kit (Promega) was used to determine luciferase activity after transfection of 48 h, with the firefly luciferase signal normalized to that of the Renilla to obtain relative luciferase activity.

### Xenograft Transplantation Experiments

The subcutaneous xenograft and peritoneal metastasis mice models were selected for exploring the proliferation and peritoneal metastasis abilities of PUM1 and DEPTOR in vivo. All mice (BALB/C nude, 4 weeks old, female) were obtained from Beijing Vital River Laboratory Animal Technology Co., Ltd. (Beijing, China). Two cell suspensions consisting of 3×10^6^ SGC‐7901 in 100 µL of PBS and 5 × 10^6^ SGC‐7901 cells in 200 µL of PBS were injected subcutaneously and intraperitoneally, respectively. The mice were sacrificed 6 weeks after the injections. Tumor samples were also collected for IHC staining. All xenograft transplantation experiments were done in compliance with Shenzhen TopBiotech Co., Ltd. (Shenzhen, China) institutional animal care regulations and conducted according to the Association for Assessment and Accreditation of Laboratory Animal Care (AAALAC) and the Institutional Animal Care and Use Committee (IACUC) guidelines (TOP‐IACUC‐2021‐0065).

### Patient‐Derived Xenograft Model and In Vivo siRNA Treatment

The PDX‐bearing mice model was raised and passaged as described previously.^[^ ^29^, ^49^ ^]^ The PDX model was initially generated using fresh tumor samples from GC patient that were subcutaneously implanted into the dorsal flank of mice as the first generation. Once an appropriate volume was reached, the tumors were excised, divided into equal pieces, and subcutaneously implanted into NOD‐SCID mice as the second generation. The 0.5 cm^3^ xenograft tumor tissues were implanted into the subcutaneous pocket on the NOD‐SCID mice for amplification of the PDX samples. When the tumor volume reached ≈150 mm^3^, the tumor‐bearing mice were randomly divided into two groups (*n* = 6 mice per group). Cholesterol‐modified PUM1 siRNA or control siRNA (10 nmol per injection, RiboBio China) dissolved in diluted water was intratumorally injected every 3 days for 15 days. The tumor volumes were calculated using the diameter and width of the tumors from PDX mice, which were measured every 3 days. All the mice were sacrificed at the appropriate time, and the tumors were removed, photographed, and weighed. All animal experiments were carried out in accordance with the NIH Guide for the Care and Use of Laboratory Animals with the approval from the Institutional Animal Care and Use Committee of Sun Yat‐sen University.

### Statistical Analyses

All in vitro experiments were repeated at least three times, and data were shown as mean ± standard deviation (SD). Quantitative data were compared using two‐tailed Student's *t*‐test and qualitative data were assessed using the *χ* ^2^ test. Pearson correlation analysis was conducted for evaluating correlations. The survival analysis was performed using the Kaplan–Meier method, log‐rank test, and Cox regression analysis. *P* < 0.05 was considered as statistically significant (^*^, *P* < 0.05; ^**^, *P* < 0.01; and ^***^, *P* < 0.001). All analyses were performed using GraphPad Prism (version 7.0), R (version 3.6.1) and SPSS statistics (version 21) software.

Additional methods can be found in the Supporting Information.

## Conflict of Interest

The authors declare no conflict of interest. All these study sponsors have no roles in the study design, in the collection, analysis and interpretation of data.

## Author Contributions

S.Y., H.L., and Z.Z. contributed equally to this work. S.Y., C.Z., Y.H., and M.L. conceived and designed the study. S.Y., H.L., Z.Z., X.X., M.H., H.W., L.G., W.C., P.W., and G.D. contributed to performing the experiments and development of methodology and writing, reviewing, and revision of the paper. S.Y., H.L., H.Y., W.C., H.W., and L.G. provided acquisition, analysis, and interpretation of data and statistical analysis. S.Y., L.Z., C.Z., and Y.H. provided technical and material support. All authors read and approved the final version of the manuscript.

## Supporting information

Supporting Information

## Data Availability

RNA sequencing data were deposited to the NCBI database under accession number GSE212848. The datasets used and analyzed during the current study are available from the corresponding author on reasonable request.
