# Supplementary material for: PUM1 Promotes Tumor Progression by Activating DEPTOR‐Meditated Glycolysis in Gastric Cancer
Source: Adv Sci (Weinh). 2023 Jul 19;10(27):2301190. doi: 10.1002/advs.202301190 (PMC10520643; doi:10.1002/advs.202301190)
Supplement: Supplementary file 1 — Supporting Information [file ADVS-10-2301190-s001.pdf]

ADVANCED SCIENCE
Open Access

Supporting Information

for *Adv. Sci.*, DOI 10.1002/advs.202301190

PUM1 Promotes Tumor Progression by Activating DEPTOR-Meditated Glycolysis in Gastric Cancer

Songcheng Yin, Huifang Liu, Zhijun Zhou, Xiaoyu Xu, Pengliang Wang, Wei Chen, Guofei Deng, Han Wang, Hong Yu, Liang Gu, Mingyu Huo, Min Li, Leli Zeng*, Yulong He* and Changhua Zhang**

Supporting Information

Title PUM1 Promotes Tumor Progression by Activating DEPTOR Meditated Glycolysis in Gastric Cancer

Songcheng Yin†, Huifang Liu†, Zhijun Zhou†, Xiaoyu Xu, Pengliang Wang, Wei Chen, Guofei Deng, Han Wang, Hong Yu, Liang Gu, Mingyu Huo, Min Li*, Leli Zeng*, Yulong He*, Changhua Zhang*

Content

Supplementary Figures (Figure S1-S6)

Supplementary Tables (Table S1-S3)

Supplementary Materials and Methods

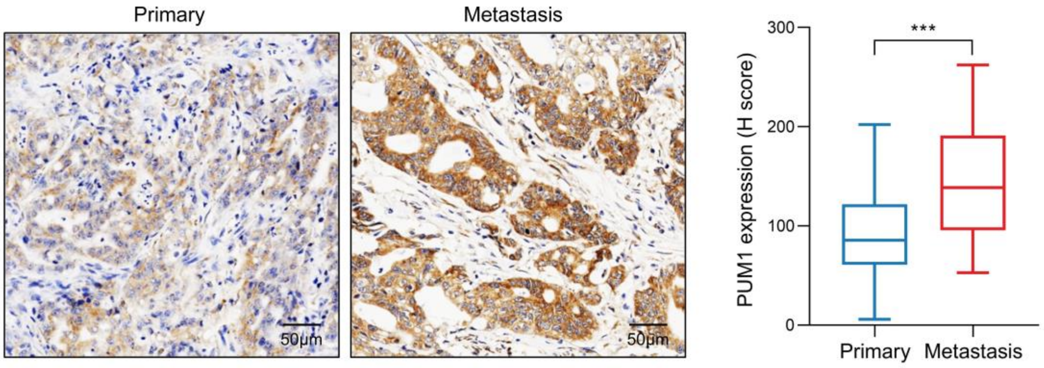

Supplementary Figure S1. Immumohistochemical staining of PUM1 in the primary and metastatic lesions of GC. Scale bar, 50 μm. The corresponding IHC scores (H-score) was compared on the right. ***, P < 0.001

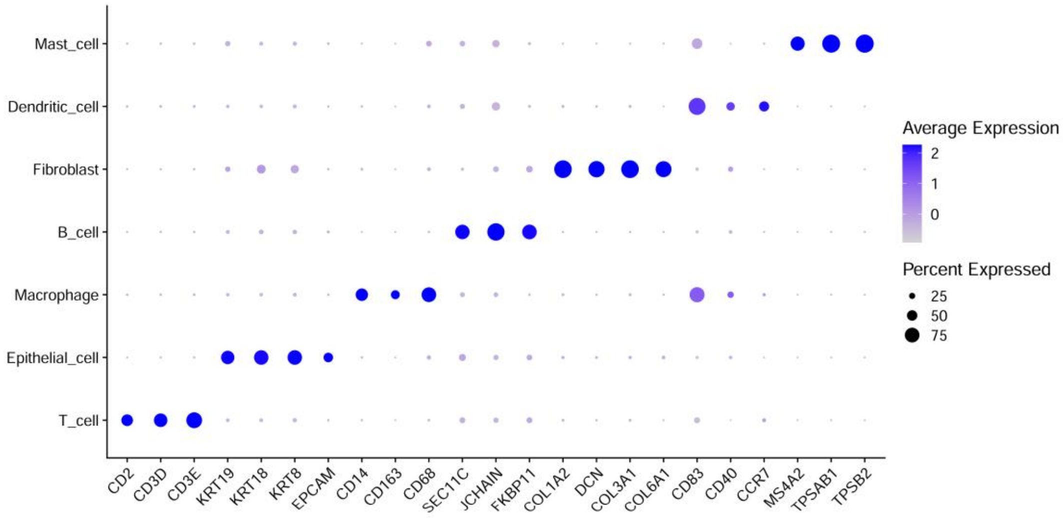

Supplementary Figure S2. Cell markers used to identify cell types in single cell sequencing analysis.

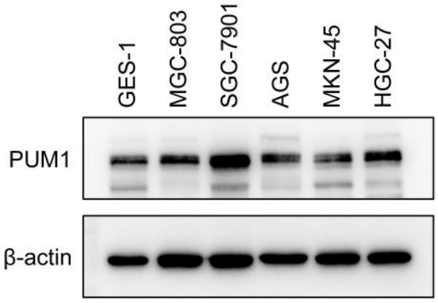

Supplementary Figure S3. Supplementary Figure S3. Expression of PUM1 in gastric mucosal epithelial cell GES1 and several gastric cancer cell lines.

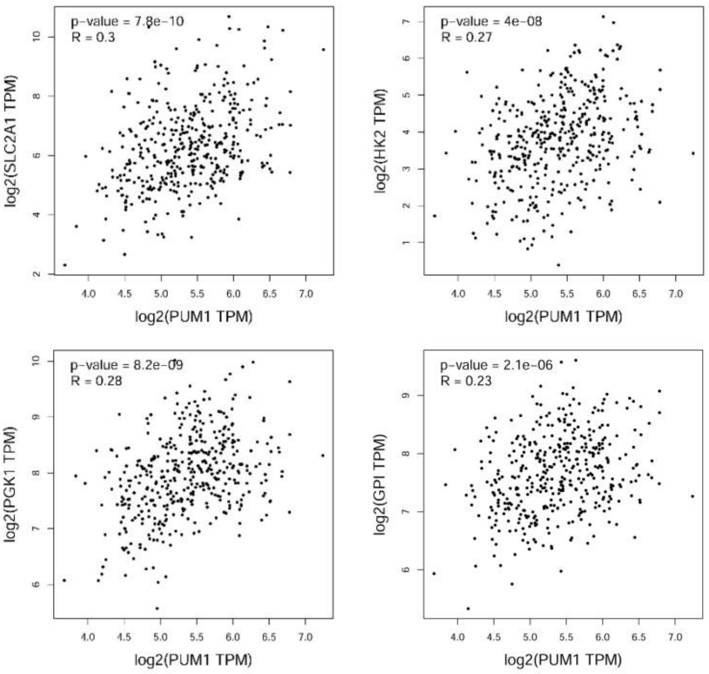

Supplementary Figure S4. Correlation between PUM1 and glycolysis-related genes expression were conducted by GEPIA platform (Pearson correlation analysis).

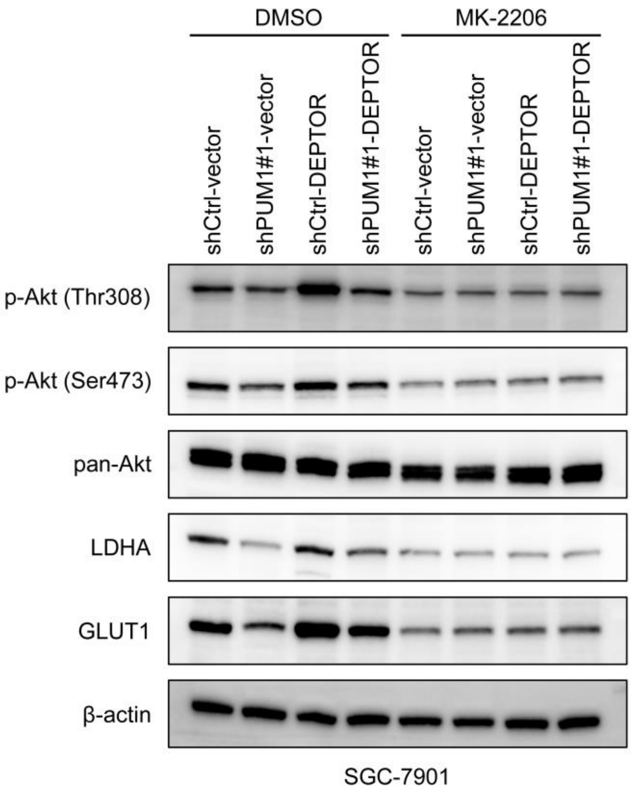

Supplementary Figure S5. PUM1 knockdown SGC-7901 cells transfected with DEPTOR constructs or empty vector control, and then treated with the Akt inhibitor MK-2206 (10 μM) for 24 h. Expression of phosphorylated Akt (Ser473 and Thr308), pan-Akt, GLUT1, and LDHA were determined by Western blot analysis.

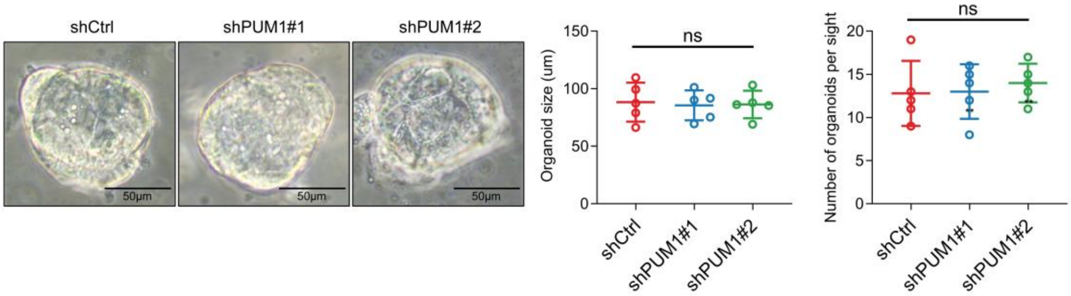

Supplementary Figure S6. The effect of knocking down PUM1 on the growth of normal gastric organoids.

Table S1. Clinicopathological characteristics and staining patterns of PUM1 in gastric cancer

Variables	Total	PUM1 expression				*P*
		High (112)		Low (136)		value
	248	No. of patients	%	No. of patients	%	
Age						0.339
≤ 60	129	62	48.1	67	51.9	
> 60	119	50	42.0	69	58.0	
Sex						0.811
Male	179	80	44.7	99	55.3	
Female	69	32	46.4	37	53.6	
Tumor size						0.020
< 5 (cm)	90	32	35.6	58	64.4	
≥ 5 (cm)	158	80	50.6	78	49.4	
Differentiation						0.386
Well/moderate	116	49	42.2	67	57.8	
Poor	132	63	47.7	69	52.3	
Depth of tumor invasion						0.025
pT1–2	61	20	32.8	41	67.2	
pT3–4	187	92	49.2	95	50.8	
Lymph node metastasis						0.001
Absent	80	24	30.0	56	70.0	
Present	168	88	52.4	80	47.6	
Lymphatic vessel invasion						0.006
Absent	156	60	38.5	96	61.5	
Present	92	52	56.5	40	43.5	
pStage						<0.001
I–II	102	31	30.4	71	69.6	
III	146	81	55.5	65	44.5	
DEPTOR						0.003
Low	119	42	35.3	77	64.7	
High	129	70	54.3	59	45.7	

Table S2. Univariate and multivariate analyses for OS in GC patients

Variable	Univariate			Multivariate		
	HR	95% CI	*P*	HR	95% CI	*P*
Age (years)						
≥60 vs. <60	1.028	0.741-1.426	0.868			
Sex						
Male vs. female	0.886	0.617-1.273	0.512			
Grade						
G3 vs. G1/G2	1.026	0.739-1.424	0.880			
Tumor size						
> 5 cm vs. ≤5 cm	2.352	1.593-3.474	<0.001	1.684	1.122-2.526	0.012
Tumor depth						
T3-4 vs. T1-2	4.700	2.599-8.502	<0.001	2.354	1.259-4.402	0.007
Lymph node metastasis						
Present vs. absent	4.948	3.012-8.129	<0.001	2.976	1.773-4.994	<0.001
Lymphatic vessel invasion						
Present vs. absent	1.243	0.891-1.735	0.201			
PUM1						
High vs. low	2.478	1.774-3.459	<0.001	1.933	1.370-2.728	<0.001
DEPTOR						
High vs. low	1.966	1.399-2.762	<0.001	1.644	1.162-2.327	0.005

Table S3. Primers sequence for qPCR

Gene	Sequence (5'-3')
PUM1-F	GCATTTGGACAAGGTCTGGCAG
PUM1-R	GCTACAAGTCGAACAGGAGCTC
DEPTOR-F	ACTGGCTGGTTCAGGAAGGTGA
DEPTOR-R	GCTGTCCACAAATGGGTGCTTG
β-Actin -F	CATGTACGTTGCTATCCAGGC
β-Actin -R	CTCCTTAATGTCACGCACGAT

Supplemental Materials and Methods

Immunohistochemistry (IHC) analysis

Tissue sections, previously fixed in formalin and embedded in paraffin, were deparaffinized using xylene prior to rehydration. Antigens were then retrieved by heating tissues for three minutes in a pressure cooker containing EDTA buffer (pH 9.0) and after blocking the samples with goat serum, they were incubated overnight at 4 °C with the primary antibodies (PUM1 1:200, Abcam, #ab92545; DEPTOR 1:100, Abcam, #ab244395; Ki-67 1:1000, Abcam, #ab15580; phospho-Akt Ser473 1:200, proteintech, #664444; HK2 1:400, proteintech, #66974; GLUT1 1:600, proteintech, #66290). A second incubation was then performed for one hour at room temperature with an HRP-conjugated secondary antibody before using the DAB Detection Kit (Polymer) (Gene Tech, #GK600510) to perform the peroxidase reaction. Based on the staining intensity, one of the following scores was given to the samples: 0 – no staining; 1 – weak staining; 2 – moderate staining; and 3 – strong staining. That score was then applied to determine a semiquantitative H-score (histochemistry score) range for each specimen by multiplying the distribution areas (0%–100%) at each staining intensity level by the intensities. Eventually, a mean H-score was selected as the cutoff value for assigning samples into high- or low-expression subgroups.

Cell lines and cell culture

The GC cell lines SGC-7901 and HGC-27 were obtained from the cell bank of the Chinese Academy of Sciences. Cell authentication was carried out by analyzing short tandem repeats before further testing the cell lines to ensure that they were free from Mycoplasma. All cell lines were cultured in RPMI 1640 medium to which 10% FBS was added prior to cell incubation at 37 °C and under 5% CO2.

Organoid culture

Human GC tissues and adjacent normal gastric tissues were taken from patients who underwent GC surgery in the Seventh Affiliated Hospital of Sun Yat-sen University for organoid preparation. After the tumor was excised, it was placed in 50 U/ml penicillin-streptomycin (Thermo Fisher) frozen G solution. The tissue was minced on ice and incubated in DMEM containing 1 mg/ml collagenase V (Sigma-Aldrich) for 1 hour at 37 °C. Iced PBS was added to stop the digestion, and subsequently centrifuged at 4 °C (300 rcf, 5 min). The sample was further digested with TrypLE (Thermo Fisher) at 37 °C for 5 min, and then stopped with a large amount of PBS. The suspension was filtered through 40 nylon meshes, centrifuged, and the cells were fixed in the matrix. It was then passaged with TrypLE every 2 weeks.

Plasmids, lentivirus production and transfection

To determine how the ability of GC cells to proliferate, form colonies, migrate and invade was influenced by PUM1 and DEPTOR, we transfected short hairpin RNA (shRNA) and overexpression plasmids or lenti-virus (constructed by GeneChem) as specified by the manufacturer. The shRNAs targeting PUM1 (targeting sequences: TTCCAAAGACCTAATGCGCTT, and CAGTTCTTTCTACGGCAACAA) were synthesized by GeneChem.

Western blotting

Lysis buffer was used for extracting proteins before their subsequent separation by sodium dodecyl sulphate-polyacrylamide gel electrophoresis (SDS-PAGE). Transfer of the separated proteins onto PVDF membranes was then followed by overnight incubation at 4 ℃ with the primary antibodies: PUM1 (1:1000, Abcam, #ab92545), DEPTOR (1:1000, CST, #11816), Pan-Akt (1:1000, CST, #4691), phospho-Akt (Thr308; 1:1000, CST, #13038), phospho-Akt (Ser473; 1:1000, CST,

#4060), HK2 (1:5000, proteintech, #66974), LDHA (1:500, proteintech, #66287), GPI (1:5000, proteintech, #67178), GLUT1 (1:1000, proteintech, #66290), PGK1 (1:500, proteintech, #17811) and β-actin (1:10000, proteintech, # 66009). Membranes were then incubated with the HRP-conjugated goat secondary antibodies before detecting the antigen-antibody reaction signal by adding the luminol-based enhanced chemiluminescence (ECL) substrate. Finally, the ChemiDoc Imaging System (Bio-Rad) was used to capture images for analysis by the Image J software (NIH, Bethesda, MD, USA; V.1.8.0).

CCK-8 cell proliferation assay

Cell proliferation was determined through the use of the Cell Counting Kit 8 (CCK-8, Biosharp) as recommended by the manufacturer's standard protocol. Cells harvested using a Trypsin-EDTA (0.25%) mixture were resuspended in 96-well plates (NEST) (2000 cells per well) before adding CCK8 reagent (10 μl) to each well at the specified time. The OD values were then measured at 450 nm after 1 hour.

Colony formation assay

This assay was also aimed at determining the cells' ability to proliferate. In this case, 1000 cells were added to each well of six-well plates and after two weeks, they were fixed with cold methanol after being washed with PBS. Finally, crystal violet was used for staining the cells.

Wound healing and invasion assay

Cells seeded into six-well plates were allowed to reach 70%–80% confluence before scratching the monolayer cells with a clean pipette tip. The effects of cell proliferation on the results were reduced by using a serum-free medium. Take photographs at the scratch of the culture plate at 0 and 48 hours, respectively, and calculate the area change of the scratch area with ImageJ software.

For the invasion assay, a 24-well plate fitted with a 8-mm PET membrane (Corning) was used. In this case, to the upper chamber which had been previously coated with diluted Matrigel (BD) (1:10 in serum free RPMI-1640 medium), a cell suspension consisting of 5×10^4 GC cells in serum free RPMI-1640 was added while to the lower one, RPMI-1640 medium containing 20% FBS was then added. After 24 h, cells present in the lower chamber were washed with PBS and after fixing the cells using cold methanol, cell staining was performed using crystal violet. For each chamber, five microscopic fields were randomly selected to determine the average number of cells.

Determination of glucose uptake and lactate production

The cells glucose uptake and extracellular lactate production were determined by using the Glucose Uptake as well as the Lactate Colorimetric Assay Kits (Biovision) as recommended by the manufacturer.

Cell cycle assay

Cell cultures, incubated overnight at 37 ℃ and in six-well plates containing complete medium, were digested with non-EDTA trypsin and collected prior to overnight fixing with 100% ice-cold ethanol. Cell staining was eventually performed with propidium iodide (Life Technologies) for 15 minutes before detecting the cell cycle of each group by flow cytometry (BD).

RNA extraction and qRT–PCR analysis

Using Trizol reagent, total RNA was extracted and subsequently reverse transcribed into cDNA. The level of mRNA expression in cells was then determined by quantitative real-time polymerase chain reaction (qRT-PCR) using 2X SYBR® Green Pro Taq HS Premix II (Rox Plus) (AG, #AG11719) as specified by the manufacturer, with β-actin selected as the internal control. Supplementary Table S3

also provides the list of primers used for the PCR.

RNA-sequencing and Bioinformatics analysis

Several independent studies in Oncomine (https://www.oncomine.org) were used along with the TCGA database (https://gdc.xenahubs.net) to analyze PUM1 expression in GC and normal tissues. For our sequencing data, differentially expressed genes (DEGs) were identified by selecting |log2 fold change < 1| and P-values < 0.05 as thresholds. In addition, by using the GSEA software, the molecular pathways correlated with PUM1 were identified based on gene set enrichment analysis (GSEA).

Single-cell sequencing analysis

The GC scRNA-seq data (GSE183904), which consisted of one normal tissue sample, 31 GC tissue samples and 11 adjacent normal tissue samples were obtained from the GEO database (https://www.ncbi.nlm.nih.gov/geo/). The single-cell analysis R package "Seurat" was employed to analyze the GC scRNA-seq data.[1] The scRNA-seq data was normalized using the "LogNormalize" method and integrated using the "IntegrateData" function. The top 20 principal components were further dimensionally reduced using the Uniform Manifold Approximation and Projection (UMAP) technique.[2] The cells were then clustered using the "FindClusters" function and annotated with typical cell markers.

Based on the median PUM1 expression level, samples were categorized into two groups: PUM1-high and PUM1-low expression groups for further analysis. Gene Set Variation Analysis (GSVA) was performed to evaluate the enrichment of hallmark gene sets in the difference gene list.[3] The hallmark gene sets were obtained from the Molecular Signatures Database (MSigDB) using R package “msigdbr”, species = "Homo sapiens", category = "H".[4]

Targeted metabolomics study

The control or PUM1 deficiency SGC-7901 and HGC-27 cells were seeded in 10-cm plates and collected using cell scrapers 24h later. The collected cells were immediately frozen using liquid nitrogen and stored in a − 80 °C freezer. The liquid chromatography–mass spectrometry (LC–MS) was performed to measure small-molecule metabolites in energy metabolism by Verygenome Technology Co., Ltd. (Guangzhou, China). The metabolite peak areas were normalized by the respective protein concentrations.

Reference

[1] A. Butler, P. Hoffman, P. Smibert, E. Papalexi, R. Satija, *Nat Biotechnol* **2018**, *36*, 411-20.

[2] E. Becht, L. McInnes, J. Healy, C. A. Dutertre, I. W. H. Kwok, L. G. Ng, F. Ginhoux, E. W. Newell, *Nat Biotechnol* **2018**.

[3] S. Hanzelmann, R. Castelo, J. Guinney, *BMC Bioinformatics* **2013**, *14*, 7.

[4] A. Liberzon, C. Birger, H. Thorvaldsdottir, M. Ghandi, J. P. Mesirov, P. Tamayo, *Cell Syst* **2015**, *1*, 417-25.
